# Targeting Lifestyle in CNS Inflammatory Demyelinating Diseases: Insights from Diet and Exercise as Potential Disease Modifiers

**DOI:** 10.3390/brainsci16010057

**Published:** 2025-12-30

**Authors:** Eleonora Virgilio, Federico Abate Daga, Matteo Bronzini, Marta Morra, Rachele Rosso, Alessandro Maglione, Manuela Matta, Federica Masuzzo, Simona Rolla

**Affiliations:** 1 Department of Clinical and Biological Sciences, University of Turin, 10043 Orbassano, Italy; federico.abatedaga@unito.it (F.A.D.); rachele.rosso@unito.it (R.R.); alessandro.maglione@unito.it (A.M.); simona.rolla@unito.it (S.R.); 2 Neurology Unit, San Luigi Gonzaga Hospital, 10043 Orbassano, Italy; matteo.bronzini@gmail.com (M.B.); marta.morra@unito.it (M.M.); m.matta@sanluigi.piemonte.it (M.M.); f.masuzzo@sanluigi.piemonte.it (F.M.); 3 Department of Computer Science, University of Turin, Corso Svizzera 185, 10149 Turin, Italy

**Keywords:** multiple sclerosis, NMOSD, MOGAD, diet, exercise, lifestyle, environmental factors, risk factors, disease progression

## Abstract

This narrative review explores the impact of diet and physical exercise both as a risk factor of central nervous system inflammatory diseases, but more importantly as potential adjunctive disease modifiers in Multiple Sclerosis (MS), Neuromyelitis Optica Spectrum Disorders (NMOSD), and Myelin Oligodendrocyte Glycoprotein (MOG) antibody-associated disease (MOGAD). The majority of evidence relies on MS preclinical and clinical studies, but preclinical studies also support the benefit of lifestyle intervention in NMOSD and MOGAD. In MS, adherence to healthy diets (particularly Mediterranean and MIND diets) could lead to a milder disease course with reduced relapse rates, while structured exercise from early disease stages promotes neuroprotection by upregulating neurotrophic factors and preserving brain volume, possibly impacting disease progression. The ketogenic diet and intermittent caloric restriction also showed promising results. Physical activity, including both aerobic training and resistance training, emerges as a potential disease-modifying strategy by promoting neuroprotection, reducing inflammation, and supporting functional and cognitive outcomes, particularly when implemented early in the disease course. A synergistic approach alongside disease-modifying treatments (DMTs) would further positively modulate core pathological processes. Evidence for NMOSD and MOGAD warrants further investigation. We highlight that integrating personalized lifestyle strategies would be beneficial from the early stages. However, future large-scale, standardized trials are required to fully confirm the neuroprotective potential of diet and exercise across the entire spectrum of CNS disorders.

## 1. Introduction

Inflammatory disorders of the central nervous system (CNS) represent a heterogeneous group of conditions, among which multiple sclerosis (MS) is the most frequent. However, in recent years, neuromyelitis optica spectrum disorders (NMOSD) and myelin oligodendrocyte glycoprotein (MOG) antibody-associated disease (MOGAD) have also gained increasing recognition [1,2]. These disorders share heterogeneous clinical features and are characterized by the progressive accumulation of disability, although they partially share common risk factors [2]. In recent years, growing attention has been directed toward the roles of lifestyle factors such as diet and physical exercise in disease development and in influencing both short- and long-term prognosis [3]. Diet and physical exercise may also affect the course of MS, NMOSD, and MOGAD through convergent effects on immune regulation, neuroglial resilience, and metabolic–inflammatory crosstalk [4]. Mechanistically, nutritional patterns and physical activity (PA) can modulate peripheral immune activation by shaping T-cell polarization (Th1/Th17 vs. regulatory T cells), B-cell function, cytokine networks (including IL-6, IL-17, and type I interferons), and oxidative stress, thereby affecting blood–brain barrier (BBB) permeability and immune cell trafficking into the CNS [5,6,7]. Within the CNS compartment, lifestyle interventions may further influence microglial activation states, astrocytic metabolic support, mitochondrial function, and neurotrophic signaling, collectively impacting demyelination, axonal injury, and repair processes [5,6,7]. While these mechanisms have been most extensively studied in MS, similar pathways, particularly complement activation, IL-6-driven inflammation, and astrocyte vulnerability, are central to NMOSD and MOGAD, providing a biologically plausible rationale for lifestyle interventions as modulators of disease activity and progression. This framework supports the hypothesis that diet and exercise act not merely as environmental exposures, but as dynamic modifiers of immune–neural interactions across the spectrum of inflammatory demyelinating disorders.

This narrative review explores the impact of diet and PA not only on disease development but also as potential disease modifiers for CNS diseases. We defined lifestyle-based disease modifiers as non-pharmacological, behavior-driven interventions implemented after disease onset that exert sustained biological effects on key pathogenic mechanisms of the CNS inflammatory diseases. Unlike risk factors or purely symptomatic measures, they are hypothesized to modulate the disease course actively. These interventions are intended to act as adjuncts and synergize with pharmacological disease-modifying therapies (DMTs).

Despite growing interest, lifestyle-based disease modifiers remain comparatively understudied in inflammatory demyelinating diseases relative to pharmacological interventions. This disparity largely reflects the intrinsic methodological challenges of lifestyle trials, including difficulties in standardization, long-term adherence, blinding, and control of multiple behavioral and socioeconomic confounders. In addition, lifestyle interventions often require prolonged follow-up to detect disease-modifying effects, are less amenable to traditional randomized controlled trial designs, and lack commercial incentives that typically drive large-scale funding. These limitations have contributed to a fragmented evidence base, underscoring the need for integrative, mechanism-informed syntheses to contextualize existing data and guide future research.

Moreover, reviews and meta-analyses [3,8] are largely MS-centric, symptom-focused, and rarely distinguish lifestyle factors as risk modifiers versus biologically active interventions. Therefore, we also extended the search to NMOSD and MOGAD, two relatively recent nosographic entities that enter the differential diagnosis with MS [1,9]. Although pathogenetically distinct, these disorders share partially overlapping immunological and neuroinflammatory mechanisms with MS, and advances in biomarker-driven diagnostics and targeted immunotherapies have significantly refined disease classification and management [1,9]. Therefore, we also included NMOSD and MOGAD in our literature search, as they represent the principal inflammatory disorders of the CNS encountered in the differential diagnosis of MS.

## 2. Methods

This narrative review is based on results from a literature search of scientific articles retrieved from several databases, including PubMed/Medline, Google Scholar, Scopus, and Web of Science. Search terms included “multiple sclerosis”, “neuromyelitis optica spectrum disorder”, “MOGAD”, “physical exercise”, “risk factors”, “diet”, “exercise”, “lifestyle”, “environmental factors”, “disease progression”, and “disease modifier”. Boolean operators (AND, OR) were also used. The final research was carried out in August 2025, providing an updated overview of the literature. Each Author independently reviewed the titles and abstracts of a specific topic, contributing to the final article selection.

## 3. Clinical and Pathophysiological Overview of CNS Inflammatory Disease

### 3.1. Epidemiology

Among the inflammatory autoimmune neurological syndromes of the CNS, MS is by far the most common. A total of 2.8 million people are estimated to live with MS worldwide (35.9 per 100,000 population) [10]. The pooled incidence rate across all locations is 2.1 per 100,000 persons/year [10], which varies depending on geographic location and ethnicity. MS predominantly affects young adults, with a higher prevalence in women and of European descent [11].

In contrast, NMOSD is a rarer entity, with prevalence estimates ranging from 0.37 to 10 per 100,000 [12]. It disproportionately affects individuals of Asian, African, and Latin American ancestry and also shows a marked female predominance. NMOSD is associated with antibodies against aquaporin-4 (AQP4-IgG), which serve as a diagnostic biomarker in the majority of cases [9,13]. NMOSD is characterized by more severe and frequent relapses with permanent neurological damage. Therefore, recovery from attacks in NMOSD is more limited compared to recovery usually seen with MS and MOGAD [14].

More recently, MOGAD has emerged as a distinct clinical and pathological entity. Characterized by the presence of MOG antibodies, it encompasses a spectrum of syndromes, including optic neuritis, transverse myelitis, and acute disseminated encephalomyelitis [1,15]. MOGAD affects both children and adults and tends to have a more balanced sex distribution. Diagnostic criteria were recently defined to improve diagnostic precision and disease classification [1,16].

### 3.2. Key Differences in Pathogenesis: Neuroinflammation and Immune Mechanisms

**Figure 1:**
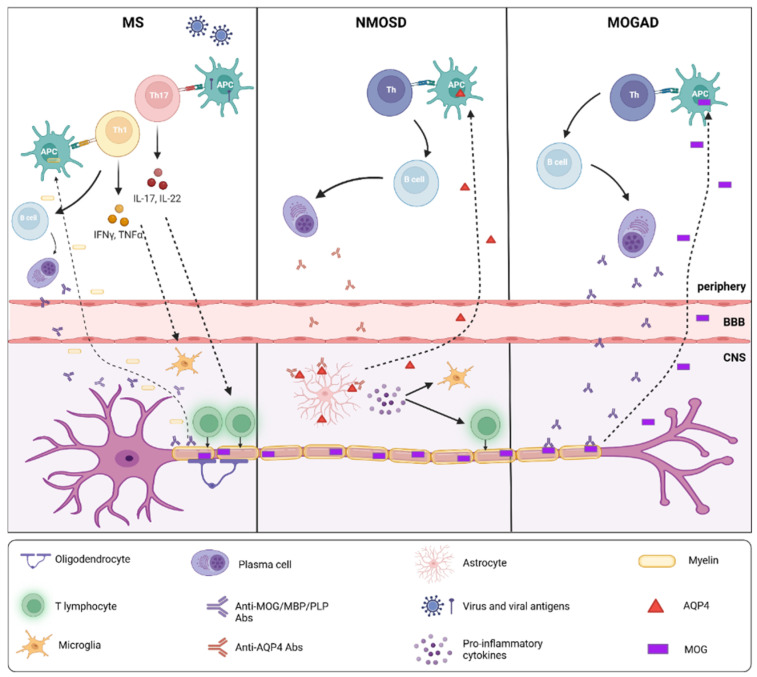
Immunopathogenic mechanisms in MS, NMOSD, and MOGAD. In MS ((**left**) panel), myelin-derived antigens such as myelin basic protein (MBP), MOG and proteolipid protein (PLP) are released from damaged myelin or oligodendrocytes and captured by peripheral antigen-presenting cells (APCs). These APCs process and present the peptides on Major Histocompatibility Complex class II molecules to autoreactive CD4^+^ T helper cells. In addition, also external triggers such as viral infections or molecular mimicry with microbial antigens may activate autoreactive T cells. Activated Th1 cells produce IFN-γ and TNF-α, while Th17 cells release IL-17 and IL-22, promoting further BBB disruption and leukocyte infiltration. The ensuing inflammatory cascade leads to microglia activation and oligodendrocyte injury and demyelination. In NMOSD ((**middle**) panel), APCs present peptides derived from the AQP4 to autoreactive Th cells. Activated Th cells support the differentiation of AQP4-specific plasma cells that secrete anti-AQP4 IgG1 antibodies. These antibodies cross the BBB, bind to AQP4 on astrocytes, and activate the classical complement cascade, leading to astrocyte destruction, microglia activation, and secondary demyelination. In MOGAD ((**right**) panel), APCs internalize and present MOG peptides to autoreactive Th cells, which in turn stimulate MOG-specific B cells to produce anti-MOG antibodies. Once these antibodies reach the CNS, they bind to MOG on the outer surface of myelin and oligodendrocytes, triggering antibody-dependent cytotoxicity and demyelination. Solid arrows represent primary direct and causative immunopathogenic interactions. Dashed arrows indicate secondary processes that modulate or amplify the immunopathogenetic processes.

From a pathogenic perspective, MS is considered a T-cell-mediated autoimmune disease, primarily targeting oligodendrocytes and leading to demyelination and axonal damage [17] (Figure 1). Autologous myelin-reactive T cells are initially primed to CNS autoantigens in the periphery and then cross the BBB [18], activate microglia and macrophages, and promote local inflammation [19]. CD8+ T cells are predominantly found at the edge and in the acute lesions, while CD4+ T cells are located deeper within the lesions [17]. In addition to the migration of autoreactive lymphocytes across the BBB, reduced regulatory T cell function has been shown to stimulate the autoimmune response in MS [20]. B cells, as well, are crucially involved in the pathogenesis of disease, through antibody-dependent and independent mechanisms [21]. They include: (i) antigen presentation to T cells and driving autoproliferation of brain-homing T cells, (ii) production of pro-inflammatory cytokines and chemokines that propagate inflammation, (iii) production of soluble toxic factors contributing to oligodendrocyte and neuronal injury, (iv) contribution to the formation of ectopic lymphoid aggregates in the meninges, and (v) providing a reservoir for Epstein–Barr (EBV) virus infection [22].

In contrast, NMOSD is characterized by a B-cell-mediated immune response directed against astrocytes, resulting in a primary astrocytopathy (Figure 1). The B-cell-mediated immune response is directed against astrocytes, leading to immune-mediated inflammation and secondary demyelination [6]. AQP4-IgG is directed against a water channel protein abundant in the astrocytic foot processes at the BBB that wraps around blood vessels and ensheathes synapses, and is highly concentrated in spinal cord gray matter, periaqueductal, and periventricular regions [23]. Penetration of the BBB and interaction with AQP4 in astrocyte feet trigger the recruitment and activation of complement, resulting in complement-dependent cytotoxicity, lysis, and loss of AQP4 expression. Complement activation also promotes the release of pro-inflammatory cytokines, including interleukin-6 (IL-6) and interleukin-17A (IL-17A), which favor a Th17-skewed immune response and contribute to the amplification and perpetuation of CNS injury [24]. The primary astrocytic damage is subsequently followed by secondary oligodendrocyte injury, demyelination, and neuronal loss, highlighting the downstream consequences of astrocytopathy in NMOSD [25]. In addition to complement-mediated mechanisms, effector immune cells, such as natural killer (NK) cells, are recruited and activated, inducing antibody-dependent cellular cytotoxicity and further contributing to astrocyte damage. [26]. The characteristic deposition of immunoglobulins and activated complement components on perivascular astrocytic processes (where AQP4 expression is highest) supports the concept that humoral autoimmunity against AQP4 is central to NMOSD pathogenesis and clearly distinguishes this disease from MS [7].

Finally, MOGAD is also B-cell mediated, but the immune attack is directed against the MOG protein (Figure 1). MOG is selectively expressed in the CNS, where it accounts for approximately 0.05% of total myelin proteins. Its location on the outermost myelin sheath layers and oligodendrocyte cell surface makes it directly accessible to MOG-IgG [15,16]. The neuropathological hallmarks of MOGAD include perivenous and confluent white matter demyelination, MOG-dominant myelin loss, intracortical demyelination, predominant CD4+ T-cell and granulocytic inflammation, complement deposition within active white matter lesions, partial axonal preservation, and reactive gliosis [5,27,28]. The contribution of complement activation in MOGAD appears to be more heterogeneous and context-dependent than in NMOSD. While complement deposition is not uniformly observed across all MOGAD lesions, some necrotizing lesions exhibit C9neo deposition, indicating complement activation and the formation of the membrane attack complex (MAC) [27]. Therefore, complement-dependent cytotoxicity can contribute to tissue injury in patients. Experimental and in vitro studies have demonstrated that MOG-IgG, predominantly of the IgG1 subclass, can activate the classical complement pathway, albeit less efficiently than AQP4-IgG, leading to oligodendrocyte and myelin damage [29]. In addition to direct complement-mediated injury, complement activation products, such as C3a and C5a, may amplify inflammation in MOGAD by promoting the recruitment and activation of granulocytes, macrophages, and CD4^+^ T cells, thereby enhancing perivenous inflammation and demyelination [30]. These aspects only partially overlap with the pathogenesis of MS. Nevertheless, the predominant intracortical location of cortical demyelinating lesions and the primary role of CD4+ rather than CD8+ T-cells and B cells in the inflammatory infiltrates distinguish MOGAD from MS. In addition, slowly expanding demyelinated plaques, a common feature in MS, are typically absent in MOGAD [15].

### 3.3. Key Differences in Pathogenesis: Neurodegeneration

Another important contributor to short and long-term disability in MS is defined by chronic inflammation and progressive neuroaxonal degeneration [31,32]. Neurodegeneration occurs early and persists, particularly in progressive forms, driven by chronic CNS-compartmentalized inflammation and the failure of remyelination [33]. Key pathomechanisms include glial activation (microglia and astrocytes) driving oxidative stress, mitochondrial dysfunction, and glutamate excitotoxicity [34]. Moreover, the formation of ectopic meningeal lymphoid follicle-like structures in MS is strongly associated with cortical demyelination and neurodegeneration, especially in progressive stages [35].

In contrast, in NMOSD, AQP4 autoantibodies, after causing primary astrocyte damage, lead to subsequent acute neuroaxonal injury [34]. Unlike MS, NMOSD pathology typically lacks extensive cortical demyelination and meningeal lymphoid aggregates, correlating with the rarity of a secondary progressive clinical course [34]. However, distinct focal cortical gray matter lesions featuring scattered neuronal loss and localized microglial activation have been described, suggesting a possible secondary neurodegeneration due to the loss of astrocyte trophic support or imbalances in glutamate homeostasis caused by defective astrocytic glutamate transporters [34]. When analyzing the potential role of neurodegeneration in MOGAD pathogenesis, several key factors must be considered. MOGAD can present with both monophasic and relapsing courses, can occur in both children and adults, and MRI lesions often tend to resolve after the acute phase [36]. Moreover, MOGAD lesions have been shown to exhibit less microstructural disruption than multiple sclerosis lesions [37]. From a radiological perspective, however, recent studies have reported worsening brain and spinal cord atrophy both during acute attack and during remission phases. In most cases, atrophy in NMOSD and MOGAD appears to be linked to clinical relapses or extensive spinal cord lesions, leading to dying-back degeneration [36]. In addition, relapsing MOGAD has been associated with reduced spinal cord cross-sectional area and lower hippocampal volumes compared with monophasic disease [36]. These findings suggest that neurodegeneration in MOGAD is predominantly secondary to acute inflammatory attacks, rather than representing an independent pathophysiological process [36]. Finally, it is important to note that acute demyelinating syndromes may negatively affect brain developmental trajectories, particularly in pediatric populations [37].

### 3.4. The Impact of the Brain–Gut Axis

A key mechanistic link between dietary patterns and CNS inflammation is represented by the gut–brain axis [4,38]. The human gut microbiome is a highly diverse community of microorganisms, encompassing more than 1000 bacterial species, as well as archaea, fungi, protozoa, and viruses. In healthy individuals, this ecosystem is largely dominated by bacteria belonging to the Bacteroidetes and Firmicutes phyla. Through a stable yet dynamic symbiotic relationship with the host, the gut microbiota plays a fundamental role in regulating immune responses and metabolic homeostasis [39]. Diet-induced alterations of the gut microbiota can influence systemic immune homeostasis by modulating intestinal permeability, microbial metabolite production, and the balance between pro-inflammatory Th17 cells and regulatory T cells [4,38]. In MS, growing evidence supports a role for gut dysbiosis in shaping autoimmune responses and neuroinflammation. Current evidence suggests that dysfunction of the gut–brain axis in MS and EAE may operate bidirectionally. On one hand, gut dysbiosis can promote a pro-inflammatory intestinal milieu and increased epithelial permeability, potentially altering afferent cholinergic signaling to the CNS and thereby amplifying neuroinflammatory processes.

On the other hand, ongoing CNS inflammation may disrupt efferent cholinergic pathways, secondarily affecting intestinal immune regulation and fostering gut inflammation as the disease evolves [38]. Although data on NMOSD and MOGAD are more limited, NMOSD patients have been shown to display a disrupted intestinal barrier, with dysbiosis and gut inflammatory activation [40]. Moreover, recent evidence has pointed to differences in many bacterial taxa in NMOSD patients, particularly Clostridium perfringens and Streptococcus, supporting a role in NMOSD pathogenesis and potentially serving as disease modifiers. It is worth noting that many studies are cross-sectional and have small sample sizes, limiting the generalizability of the results. To date, the gut microbiome has yet to be investigated in a MOGAD cohort; however, a newly identified Erysipelotrichaceae bacterium, when co-cultured with Lactobacillus reuteri (which shares homology with MOG peptides), was shown to enhance MOG-specific Th17 responses and worsen EAE severity [41].

## 4. Environmental Risk Factors and Pathogenetic Differences

The pathogenic divergence among MS, NMOSD, and MOGAD may reflect differences in ambient risk factors and modifiers, which in turn could influence differences in lifestyle-based interventions, such as diet, exercise or stress management. In the following section, we will discuss environmental factors associated with a higher risk of inflammatory CNS diseases, except for diet and physical activity (PA), which will be addressed separately. To improve readability and facilitate cross-disease interpretation, environmental factors are presented separately for the three disorders. Where available, evidence is discussed comparatively across MS, NMOSD, and MOGAD, highlighting convergent and divergent patterns.

### 4.1. Multiple Sclerosis

MS is a complex disease for which risk gene variants, such as the presence of HLA-DRB1*15:01 and the absence of HLA-A*02, as well as environmental factors, are both critical [42,43,44]. Those later include infectious agents, exposure to tobacco, obesity in adolescence, dietary factors, limited sun exposure with consequent low vitamin D, and possibly night shift work [43,44].

Among infectious agents, Epstein–Barr Virus (EBV) has the most consistent evidence [45,46]. Prospective cohort data, most notably from large studies of US military personnel, demonstrate that EBV seroconversion precedes virtually all MS onsets, with a 32-fold increased risk of MS following EBV infection and almost no cases of MS in EBV-seronegative individuals. This provides compelling epidemiological evidence for a causal relationship between EBV and MS [45,46,47]. Moreover, Human Leukocyte Antigen (HLA) risk genes and environmental factors have been observed to interact synergistically to increase the risk of MS. As HLA risk alleles encode molecules that regulate adaptive immunity, interaction with measures of EBV infection may reveal common pathogenic pathways triggering MS [42,45,46,48]. One of these could be the phenomenon of “molecular mimicry,” where parts of the virus elicit an autoimmune response directly against the CNS component, or even a direct immune attack on the virus, collaterally damaging the CNS [42,45,46,48].

Meta-analysis and cohort studies confirmed a clear dose–response relationship in which the cumulative dose of smoking is related to the risk for MS [43,49,50]. Exposure to smoke has also been associated with increased risk of progression and brain atrophy [49,50,51,52]. Cigarette smoke initiates a proinflammatory response in the lungs, promoting oxidative stress and altering self-antigens, thereby generating neoantigens [49,50]. This inflammatory milieu primes autoreactive T-cells that can subsequently traffic to the CNS, where they participate in the development of MS. The lungs thus serve as an activation site for these T cells, while smoking-related molecular alterations disrupt the BBB, facilitating their entry into the CNS [49,50]. Additionally, smoking shows a remarkable interaction with MS-associated HLA risk genes: smokers carrying HLA MS risk genes display a higher risk of MS than those without these genes [42,44]. Smoking also boosts the probability of developing neutralizing antibodies against disease-modifying treatments (DMTs), such as Natalizumab [53] and Interferon β [54].

Vitamin D and risk of inflammatory diseases have also been extensively studied. Different studies have investigated the effects of Vitamin D on human adaptive immune cells [55,56]. Vitamin D prevents B-cell proliferation and differentiation and reduces Ig secretion. It also affects T-cell maturation, leading to reduced Th1 and Th17 differentiation [56]. Vitamin D also has myelin-protective effects independent of T-lymphocyte activation and infiltration. High Vitamin D supplementation was associated with weaker microglial activation and macrophage infiltration in the white matter during oligodendrocyte death and demyelination in the animal model [57,58]. A decreased risk of MS has been observed with increasing vitamin D levels, especially before the age of 20 [59]. Additionally, a diet rich in vitamin D, including fatty fish, also reduces the risk of MS in individuals with low sunlight exposure [60].

Adolescent lifestyle characteristics, such as diet, physical activity, and obesity, are particularly relevant because they may influence disease risk in adult life and long-term progression. Childhood and adolescent obesity has been consistently associated with an increased risk of multiple sclerosis, with epidemiological studies reporting approximately a twofold higher risk in both pediatric-onset and adult-onset MS. [43,61]. Obesity is associated with a persistent low-grade inflammation characterized by increased levels of proinflammatory mediators (including leptin, resistin, and visfatin) produced by adipose tissue [43,61]. Leptin, a hormone with cytokine-like properties predominantly secreted by adipocytes, represents a key link between obesity and immune dysregulation. Circulating leptin levels correlate positively with body weight, body fat percentage, and BMI in both MS patients and obese individuals [62,63,64]. This dysregulated adipokine profile drives the polarization of Th1 and Th17 cells, promotes the activation of M1 macrophages, and impairs the suppressive activity of regulatory T cells, thereby fostering autoimmunity and inflammation within the CNS [61]. In contrast, body mass index at the time of MS diagnosis does not appear to significantly influence disease susceptibility, suggesting that early-life metabolic exposures may be particularly relevant. The biological mechanisms underlying this association are still under investigation, but are thought to involve immune–metabolic interactions mediated by adipokines and alterations in the gut microbiota [64]. In parallel, obesity-related modifications of the gut microbiota have been identified as additional contributors to immune imbalance. Notably, similarities between the pro-inflammatory microbial profiles observed in obese individuals and those reported in MS patients have been described, supporting the hypothesis that early metabolic and microbial perturbations may converge on shared inflammatory pathways. Together, these findings suggest that obesity during critical developmental windows may prime the immune system toward a sustained pro-inflammatory state, thereby increasing vulnerability to autoimmune demyelinating disease later in life [61,64].

Night shift work and sleep deprivation have been explored as potential risk factors for MS, with inconsistent results. In two large cohorts of nurses, no overall association was observed between rotating night-shift work and MS risk. However, other studies found that individuals who started shift work before age 20 had an increased risk (OR 1.5), whereas the association was weaker among those who began later [65]. Evidence suggests that early-life exposure may be particularly relevant. A strong association between shift work at a young age and MS risk, independent of the latency between exposure and disease onset, was reported in Sweden, and a Danish study confirmed a significant association for shift work during adolescence (ages 15–19 years) [66,67,68]. Sleep characteristics in adolescence also appear relevant. Short sleep duration (<7 h/night) and poor subjective sleep quality were associated with an increased risk of MS, while circadian phase shift was not. These associations persisted after excluding individuals with a history of shift work, indicating an independent effect of sleep deprivation. [65,66,67,68,69].

### 4.2. NMOSD and MOGAD

Since NMOSD and MOGAD pathogenesis differ from MS, some evidence suggests that distinct environmental factors may influence their development. NMOSD appears to be more common in the Asian and Black population compared to White individuals [70]. The basis for the increased risk of NMOSD in certain ethnic groups is not fully understood, but may be due to both genetic and environmental factors. Certain HLA variants, particularly those found in specific ancestral groups, may increase the risk of NMOSD. For instance, HLA-DPB105:01 and HLA-DRB116:02 are shared by Chinese and Japanese populations and are associated with increased NMOSD susceptibility [70]. Additionally, higher genetic susceptibility has been observed in Native Americans in Mexico and in HLA-DRB1 variants among Muslim Arabs [2,70]. A nationwide case–control study conducted in Canada identified a significantly higher risk of NMOSD among immigrants compared with individuals born in Canada [70]. This observation is particularly relevant, as it contrasts with the documented data on MS in the same setting [70]. Such divergent patterns may point to fundamental differences in environmental exposures or gene–environment interactions that contribute to the pathogenesis of NMOSD and MS.

As with other autoimmune diseases, a direct association between long-term smoking and antibody positivity in patients with NMOSD was observed. Smoking can damage astrocytes, thereby altering AQP4 expression and, consequently, antibody development [2,71]. It also increases the level of T-helper 17 cells in the blood and the number and activity level of CD8+ T cells [72]. To note, findings regarding the influence of smoking varied across ethnic groups, with inconsistent results reported among Caucasian and non-Caucasian cohorts [2]. Nonetheless, smoking cessation should be recommended in patients with NMOSD, possibly influencing the relapse rate.

Regarding Vitamin D and NMOSD, Shaygannejad et al. were the first to report an association between vitamin D and AQP4-IgG serostatus, suggesting that vitamin D may significantly influence antibody production in NMOSD patients and play a crucial role in the pathogenesis [73]. This association was further examined by subsequent studies, mostly conducted in Asian countries. Among East Asian populations, vitamin D deficiency appeared to increase NMOSD susceptibility; however, other studies conducted in Sri Lanka and Indonesia yielded conflicting results [2]. Even though evidence for vitamin D deficiency in NMOSD is inconsistent across populations, vitamin D may, mechanistically, mitigate astrocytic oxidative stress and complement activation [56,74,75]. Deficiency in vitamin D has been linked to aberrant activation of the complement system, characterized by altered levels of complement factors B and C9 (two critical components of the complement cascade) [75]. Human proteomic studies have shown that reduced serum concentrations of 25-hydroxyvitamin D are associated with dysregulated expression of complement proteins and heightened complement activity, supporting a regulatory role of vitamin D in modulating innate immune responses and inflammatory processes [56,75,76]. Finally, a recent systematic review comprehensively examined 50 environmental determinants of NMOSD, highlighting significant associations with Mycobacterium avium paratuberculosis infection, smoking, vitamin D, and certain dietary habits, which will be discussed later [2].

For MOGAD, the role of lifestyle interventions is less defined. However, given its autoantibody-mediated nature, interventions aimed at maintaining systemic immune balance and reducing environmental triggers (e.g., infections, stress) may help prevent relapses or optimize recovery. Plus, the disease can be recurrent or monophasic, leading to a high clinical heterogeneity. Recent multicenter cohorts demonstrate that MOGAD smokers have a higher risk of residual disability after the first attack compared with non-smokers [77,78]. This supports a model in which systemic toxic exposure aggravates acute immune-mediated demyelination. Plus, an association with obesity was reported in one study [79]. Other proposed risk factors, particularly relevant for pediatric patients, include recent infections and vaccinations [78].

## 5. Diet and Nutrition: From Risk Factors to Potential Disease Modifiers

Over the last decades, research on lifestyle and diet has suggested that different dietary patterns may influence the gut microbiota and the gut–brain axis, acting as both risk factors and modifiers of disease course in MS and related disorders. Anti-inflammatory diets, PA, and stress reduction can modulate immune function, possibly delaying progression or reducing relapse rates in MS [3,4]. These interventions may influence T-cell phenotypes and reduce oxidative stress, both of which are crucial to MS pathogenesis [3,19,80]. Diet may not only represent a risk factor for the development of CNS inflammatory disease, but can also act as a potential modifier of its course, either worsening or improving the progression or relapse risk of MS, NMOSD, and MOGAD. Both micronutrients and macronutrients were investigated as risk factors and modifiers of MS in preclinical and human models [4,81,82]. Macronutrients (carbohydrates, proteins, and lipids) and micronutrients (vitamins and minerals) strongly influence the composition and function of the gut microbiota, thereby modulating the gut–brain axis through multiple pathways. Diets rich in fiber and complex carbohydrates promote short-chain fatty acid (SCFA)–producing bacteria, such as butyrate, propionate, and acetate, which regulate intestinal permeability, immune responses, and neurotransmitter production, exerting anti-inflammatory and neuroprotective effects.

In contrast, Western diets high in saturated fats and animal proteins and low in fiber promote dysbiosis, increase pro-inflammatory metabolites, and are associated with chronic inflammation and mood disturbances. Protein-derived metabolites, including tryptophan-derived indoles, influence serotonin synthesis and brain function. Lipids, particularly omega-3 fatty acids, support beneficial microbial profiles and neuroprotective metabolite production. Micronutrients such as vitamin D, iron, zinc, and magnesium contribute to microbial regulation, immune modulation, and neurotransmitter synthesis, while deficiencies may impair microbial diversity and increase susceptibility to neuropsychiatric disorders [4,81,82]

### 5.1. Impact of Micronutrient in EAE

Certain nutrients in the human diet that are consumed in excess due to their high palatability, such as salt, sugar, and saturated fats, when added at high doses to the mouse model, promote Th17 cell differentiation and exacerbate EAE [83,84,85]. Conversely, mice fed diets rich in fiber or isoflavones have been reported to show protection against the disease, modulation of the gut microbiota, and effects on the immune response [86,87].

A growing body of research indicates that elevated salt levels can influence the differentiation, activation, and functional responses of various immune cell populations [88]. Data from preclinical studies suggest that a high-salt diet suppresses the development of experimental autoimmune encephalomyelitis (EAE). This protection could be mediated by elevated serum glucocorticoid levels [89]. Corticosterone tightens the BBB by enhancing tight junction protein expression (ZO-1 and claudin-5), thereby controlling T cell entry into the CNS and limiting disease onset [89]. On the contrary, excessive dietary salt intake seems to worsen disease severity during the acute phase, although this effect is not sustained in the chronic stage. In these models, high salt intake selectively promoted neutrophil infiltration into the spinal cord without significantly altering the recruitment of T cells, B cells, or dendritic cells. Notably, pharmacological inhibition of neutrophil migration alleviated both clinical deterioration and microglial activation induced by a high-salt diet. Similarly, treatment with minocycline, suppressing microglial activation, led to substantial clinical improvement in EAE mice exposed to elevated salt levels [90]. However, current evidence does not allow a definitive conclusion that a high-sodium diet worsens or accelerates the progression of MS patients. Other micronutrients, in particular vitamins, were also studied in the EAE animal model and in MS patients. Vitamins may influence MS pathophysiology by modulating immune responses, limiting oxidative stress, and supporting neuroprotective mechanisms [91,92]. Vitamin D is the most studied and, beyond its immunoregulatory effects, plays a role in supporting neuronal survival by attenuating pro-inflammatory cytokine production and enhancing the expression of neurotrophic factors. In both immune cells, including B and T lymphocytes, and CNS-resident cells such as oligodendrocytes, neurons, and microglia, vitamin D exerts its biological actions through binding to a specific intracellular receptor [91,92,93]. The resulting vitamin D–receptor complex regulates the transcription of target genes involved in immune modulation and neuroprotection [93]. Vitamin A also showed consistent immunomodulatory effects in EAE, whereas findings related to B, C, E, and K vitamins remain more variable and less conclusive [94]. Vitamin A and its active metabolite, retinoic acid, contribute to processes of neural regeneration and plasticity, promoting immune tolerance by modulating T-cell, B-cell, and dendritic cell function. In this context, vitamin A deficiency has been associated with impaired immune regulation and a shift toward pathogenic immune responses promoting EAE development [94].

### 5.2. Micronutrients as a Disease Modifier in Multiple Sclerosis

Among micronutrients, vitamin D is by far the one with the most consistent evidence as a disease modifier in MS. Several studies have evaluated vitamin D supplementation, reporting encouraging outcomes and reductions in fatigue and relapse rates among participants [95,96]. During beta-interferon trials, higher vitamin D levels were associated with lower MRI activity and slower disease progression [97]. Moreover, longitudinal studies in patients with RRMS further demonstrated that higher serum vitamin D levels were inversely associated with the risk of disease exacerbations, with the highest concentrations associated with the lowest relapse rates [98]. These findings were reinforced by a systematic review of vitamin D research published between 2005 and 2015, which identified potential therapeutic benefits, including fewer relapses, reduced new brain lesions, and an improved inflammatory profile [99]. Nevertheless, differences in dosages and treatment durations across the studies limited comparability and precluded firm conclusions regarding vitamin D efficacy. Finally, high vitamin D levels are associated with reduced axonal injury, as measured by cerebrospinal fluid neurofilament light chain [100], a fluid biomarker linked to relapse, inflammatory activity, and patient progression [101]. Additionally, several studies have investigated the role of vitamin D in cognitive processes, and MS patients with more pronounced Vitamin D deficits have been found to exhibit impaired information-processing speed [93].

Finally, in MS patients, observational studies suggest that greater sodium intake may be associated with increased disease activity, including higher relapse rates and more frequent development of new lesions in patients with relapsing–remitting (RR) MS compared with those consuming less sodium [102]. However, a large prospective study with a 5-year follow-up found no association between sodium intake (estimated through urinary excretion) and clinical progression or radiological disease activity [103].

### 5.3. Macronutrients and Dietary Habits in EAE and MS

A galactose-rich diet significantly exacerbated EAE severity and impaired recovery in mice [104]. This effect was driven not only by acute inflammation but also by enhanced neurodegeneration. The detrimental effect is proposed to be mediated by the formation of advanced glycation end products, which increased in the spinal cord of mice and promoted oligodendrocyte and neuronal cell apoptosis in vitro. Thus, galactose and its reactive products could be potential disease modifiers in MS [104].

In recent decades, diets high in saturated fats and animal proteins and low in fiber have been studied for the potential to enhance chronic and sustained dysbiosis and inflammation, possibly contributing to the development of autoimmune disorders and influencing their natural history [105]. In the context of MS, specific macronutrients could impact both relapse rate and neurodegenerative patterns.

Higher fat intake, especially saturated fat, significantly increased the risk of relapse (a 10% increase in total fat energy raised the hazard by 56%). In contrast, increased vegetable intake was independently protective, reducing the risk of relapse by 50% per additional cup-equivalent. These findings suggest high-fat intake may be detrimental, while vegetables may be protective in pediatric MS [106]. A subsequent study in the same US network of pediatric MS centers reported that a 50% increase in dairy intake above the recommended level was associated with a 41% increase in relapse risk and a 40% increase in T2 MRI activity [107].

These observations have been further supported by prospective studies demonstrating a protective role of vegetable-rich diets and a detrimental effect of high saturated fat intake on the clinical course of pediatric MS [107,108].

In a cohort study in southern Europe, dietary fiber intake and alpha-linolenic acid intake were inversely correlated with the risk of a first demyelinating event. [95]

In parallel with the contribution of individual micro and macronutrients, the research also investigated the possible association between dietary habits and MS. Evidence comes from different parts of the world. It includes both pediatric and adult-onset MS [43]. An older Canadian study found that consuming vegetables, fruit, and cereals had a protective effect, while diets with higher energy intake (OR 2.03) and animal fats (OR 1.99) were associated with an increased risk of adult-onset MS [109]. A subsequent study in Saudi Arabia found that consuming fast food more than five times a week was associated with an increased risk (adjusted OR 2.05), whereas eating at least five portions of fruit per week and drinking coffee daily reduced the risk [110]. In Australia, the Ausimmune Study described that a healthy diet was associated with a 25% reduction in the risk of a first clinical diagnosis of CNS demyelination [111]. It was also reported that a higher intake of nitrates from plant foods and vegetables, but not from other sources, was significantly associated with a lower probability of a first clinical event in females [112]. A recent meta-analysis of Australian and Middle Eastern studies examined the relationship between a pro-inflammatory diet and the risk of developing MS or other demyelinating inflammatory diseases. The results revealed a significant increase in the OR of MS/autoimmune demyelinating [113]. Nevertheless, an American study of the Nurses’ Health Study I and II cohorts found no association between healthy dietary quality indices and MS risk. The study authors note that it is impossible to rule out the possibility that an inverse association between better diet quality and MS risk may exist in populations with healthier dietary habits than those in the NHS cohorts used [114].

A 2021 trial of 100 people with MS showed that an anti-inflammatory diet, compared with a healthy control diet, lasting 12 weeks, significantly increased IL-4 levels without altering IL-17 or hs-CRP levels. These effects were associated with improvements in fatigue and quality of life (QoL) [115]. The authors commented on this result regarding IL-4 levels, suggesting that they may be a possible effect of anti-inflammatory dietary components, which suppress Th1 cells and increase Th2 cell activity [115].

In a long-term follow-up cohort study, healthy diets at 5 and 10 years were associated with lower changes in periventricular lesion load, but not in the iuxtacortical region or in combined areas [116]. These data are consistent with a previous study, which showed that MS patients with less healthy dietary preferences (lower dietary scores) had a higher accumulation of T2-lesion volume during the 5-year follow-up period [117]. The limitations of the studies mentioned are the observational nature of the experimental design, which prevents a clear causal link from being established.

Most available data suggest that dietary habits may affect MS prevention and disease course. Given that increases in unhealthy nutritional patterns are outpacing those in healthy patterns in most regions of the world, it is essential to implement policies that improve diet quality globally to prevent immune-mediated diseases such as MS [118,119].

### 5.4. Dietary Patterns as Disease Modifiers in Multiple Sclerosis

Research into the role of diet as a potential disease modifier is recent but encouraging. Individuals with MS often show a strong interest in modifying their diet as a possible strategy for managing the disease and enhancing their sense of control. Qualitative studies reveal that many experience uncertainty about where to obtain trustworthy nutritional guidance, express doubts about general dietary recommendations, and demonstrate a clear interest in MS-specific nutritional advice. Patients frequently implement individualized dietary approaches, evaluate their effectiveness based on perceived symptom changes, and emphasize the importance of accessible, evidence-based dietary information tailored to MS [120,121]. Several lines of evidence support the role of diet as a disease modifier in both EAE and humans.

#### 5.4.1. Ketogenic Diet

When evaluating the impact of a specific diet, the ketogenic diet (KD), a high-fat, low-carbohydrate dietary regimen, has been shown to prevent motor deficits, reduce clinical scores, inhibit demyelination, improve pathological lesions, and suppress microglial activation in the spinal cord of EAE mice [122]. In a 6-month prospective study of KD in MS patients, a decrease in self-reported fatigue and depression scores was reported after the intervention period. Significant improvements were also observed in EDSS scores, 6 min walk, and Nine-Hole Peg Test scores. In addition, leptin levels decreased, while adiponectin levels increased [123]. The same working group demonstrated that 6 months of KD maintained low, stable levels of serum neurofilaments, confirming its safety profile in MS. In addition, patients with higher serum levels of ketone bodies during the diet had improved serum neurofilament light chain levels compared to those with lower ketone body levels. This suggests that the degree of ketosis correlates with the attenuation of neuroaxonal damage in MS patients [124].

#### 5.4.2. Caloric Restriction

Reducing daily calorie intake induces hormonal, metabolic, and cytokine alterations that may be beneficial for autoimmune disease. Caloric restriction has been linked to reduced clinical severity of EAE [125,126,127], and based on preclinical evidence, two recent studies have evaluated the effect of intermittent calorie restriction (iCR) on the immune system in MS patients. An 8-week randomized controlled trial assessed the impact of 5:2 iCR (100% of their caloric needs for 5 days a week and 25% of their caloric needs for 2 days a week) compared to standard calorie restriction (CR, 78% of their caloric needs 7 days a week) or a standard diet (100% of their caloric needs 7 days a week). In the iCR group, a reduction in memory T cell subsets and Th1 was observed at the 8-week follow-up. Additionally, changes were observed in the products of glycerophospholipid metabolism that could mediate alterations in immune cell subsets [125]. Finally, no changes in serum leptin or adiponectin levels were observed in any of the CR diets during the 8 weeks. The second study evaluated the effect of iCR in a 12-week randomized controlled clinical trial [126]. The iCR group had calorie restriction to 500 kcal for 2 non-consecutive days per week, while the control group had a free diet. Similarly, naive CD4+ T cells and Th1 cells were reduced during the trial in patients following the iCR, Treg were more active, whereas leptin levels were significantly lower. Clinically, an improvement in information-processing speed was observed in MS patients [126]. We could speculate that the partial differences in leptin variation between the two studies may reflect the different periods of investigation (8 weeks versus 12 weeks).

#### 5.4.3. Mediterranean Diet

The Mediterranean diet (MD) is one of the most extensively studied dietary patterns in MS. In a comparison of different dietary types, a systematic review and network meta-analysis found that the MD showed greater reductions in fatigue compared to the control diet [127]. The MD showed greater improvements in physical and mental QoL than the control diet [127]. Plus, in MS patients, a reduction in body mass index and a slight, but statistically insignificant, decrease in fatigue scores, were reported following MD [128]. The degree of adherence to the MD has also been reported to be inversely associated with disability in several studies using the EDSS, MSSS, and Multiple Sclerosis Walking Scale [4,129,130,131,132]. Furthermore, one study showed that higher MD adherence scores mitigate the negative impact of disease duration (calculated as greater than 14 years), as measured by the MS Functional Composite [132]. In contrast, an Iranian study found no association between a higher MD score and lower frequencies of disability severity [133].

#### 5.4.4. MIND Diet

It was recently reported that early-diagnosed MS patients benefit from the Mediterranean-DASH Intervention for Neurodegenerative Delay (MIND) diet, particularly in preserving thalamic volumes, which is a crucial brain area involved early in neurodegenerative processes in MS [134,135]. The MIND diet is a hybrid of the MD and DASH diets, specifically designed to reduce the risk of neurodegenerative disease, emphasizing consumption of green leafy vegetables, berries, nuts, beans, whole grains, seafood, poultry, olive oil, wine, and limited intake of animal and high-saturated-fat foods [136]. In a case–control and longitudinal study with a 12-week dietary intervention testing the MIND diet, a significant reduction in fatigue and an increase in physical QoL were reported in the MS cohort, but not in mental QoL. In addition, a reduction in BDNF levels was described after the nutritional intervention, suggesting a possible effect of the diet in antagonizing inflammation in the glial response to neuronal damage. Finally, a statistically significant decrease in biomarkers of oxidative stress, was observed. Oxidative stress plays a role in the incorporation of essential fatty acids into cell membranes; therefore, the MIND diet could help reduce glial cell membrane damage, which is related to the aetiopathogenesis of MS [137]. Confirming these data, the results of a nutritional intervention by another group show that after 8 weeks of the MIND diet, the serum level of total antioxidant status increased significantly at the end of the study compared to baseline, and there was no significant change in total serum oxidant status and malondialdehyde levels [138]. Based on these initial findings, the MIND diet, which emphasizes green leafy vegetables, whole grains, legumes, nuts, and berries, appears to influence oxidative stress and BDNF levels in people with MS. Most RCTs on diet have so far assessed the impact on quality of life (QoL) and fatigue. A meta-analysis of clinical trials up to December 2021 showed that dietary interventions are associated with a trend towards reduced fatigue and increased QoL. Conversely, the evidence accumulated in this meta-analysis is insufficient to support the claim that disease-related disability, as measured by the EDSS, is modified by dietary changes [139].

#### 5.4.5. Low-Fat, Paleolithic, and Modified Paleolithic (Swank and Wahls) Diets

Other dietary approaches investigated in MS include low-fat and Paleolithic-based regimens. The Swank diet is a low-fat regimen that restricts saturated fat intake to no more than 15 g per day, while allowing up to 50 g of unsaturated fat daily. This eating plan emphasizes whole grains, fruits, vegetables, and lean protein sources, while limiting red meat, dairy products, and highly processed foods [8]. This regimen [3] has been associated with improvements in fatigue and, in some reports, cognitive performance [3,8]. The proposed mechanisms involve modulation of lipid metabolism, inflammatory pathways, and myelin integrity. The Modified Paleolithic Diet Intervention, also known as the Wahls Diet, prioritizes micronutrient-rich foods and recommends consuming at least 9 cups of vegetables daily, including leafy greens, sulfur-containing vegetables, and deeply pigmented produce [3,8]. The diet additionally encourages lean protein sources, nuts, and healthy fats, while excluding gluten, dairy products, and processed foods. The potential health benefits are attributed to the high intake of antioxidants, omega-3 fatty acids, and essential vitamins, which may help regulate neuroinflammatory processes and reduce oxidative stress [3,8]. However, long-term clinical evidence of their potential as disease modifiers remains limited for both diets. Additionally, concerns about potential vitamin and micronutrient deficiencies associated with prolonged adherence to the diet should be noted [3,8].

#### 5.4.6. Diet Quality, Cardiovascular Risk, and Disease Outcome

Patients with cardiovascular comorbidities are known to have greater progression rate and cognitive impairment. Therefore, some studies focused on evaluating dietary scores to estimate cardiovascular risk also in MS patients. Several studies observed internationally and prospectively a lower disability status as well as relapse rate in MS patients with lower cardiovascular risk [140,141,142,143,144,145]. However, other studies showed conflicting results [146]. An association between diet quality and the number of relapses at 5 years was observed in the AusLong Study [63], with higher scores on the Prudent diet model (a fat- and cholesterol-controlled diet). The above-mentioned relapse rate data should be interpreted with caution, given the lack of adjustment for DMTs adherence in these studies [140]. Table 1 reports a summary comparing effects of all different diets in MS.

### 5.5. Diet and Nutrition in NMOSD and MOGAD

Some investigations have identified nutritional factors such as high sugar intake, a high-carbohydrate diet, lower intake of whole grains and legumes, and a high carbohydrate-to-protein ratio as possible modifiable risk factors for NMOSD. Inflammation could result from an imbalance between antioxidant levels and free radical levels. Higher dietary total antioxidant capacity can help suppress free radicals and protect the body from inflammatory disorders [147]. Diet and NMOSD risk were also investigated by some studies, particularly in Iran [148,149,150,151,152,153], which examined consumption of a pro-inflammatory diet (defined by a validated 168-item food frequency questionnaire. A higher risk of NMOSD development was associated with dietary total antioxidant capacity (TAC). The higher the TAC, the lower the NMOSD risk, reflecting a protective role of antioxidants. In another study, researchers found that lower protein and fat intake, combined with higher carbohydrate intake, may be associated with an increased risk of developing NMOSD. After adjusting for age, sex, body mass index, and energy intake, a higher score on a low-carbohydrate diet was associated with a significantly lower OR for NMOSD and lower sugar intake. Moreover, individuals who consumed higher amounts of whole grains and legumes had a substantially lower risk of developing NMOSD than those with lower intake [149,150,151,152]. Dietary habits may play a particularly important role during adolescence. A higher risk of developing NMOSD was observed among individuals with lower intake of dairy products, seafood, red meat, eggs, chicken, fruits, vegetables, and dietary fats [148]. These findings suggest that inadequate consumption of nutrient-rich foods during this critical developmental period may increase susceptibility to NMOSD. Specifically, an inverse, dose-dependent association was observed between whole-grain or legume intake and NMOSD odds. Those data are particularly important, since legumes provide soluble and insoluble fibers, resistant starch, and oligosaccharides, which contribute to a healthy gut microbiome by stimulating the growth of Lactobacillus and Bifidobacterium [151]. Similarly, adherence to a Mediterranean dietary pattern, characterized by a high intake of dietary fiber, was associated with a more favorable gut microbial composition. Whole grains are another important source of indigestible fibers that undergo fermentation by gut microbiota, leading to the production of short-chain fatty acids. Notably, nearly 40–50% of patients enrolled in those studies were AQP4-IgG negative; however cluster analysis based on AQP4 serostatus confirmed the results [2]. High prevalence of overweight and obesity in NMOSD and high consumption of a pro-inflammatory diet in NMOSD using the dietary Inflammatory Index was also confirmed in a small cohort of Brazilian patients [149].

Regular use of nutritional supplements such as multivitamins, iron, vitamin B12, and vitamin C, possibly unraveling that micronutrient deficits could have a role in the development of autoimmunity, has also been associated with a lower likelihood of developing NMOSD [2]. In contrast, the intake of calcium, folic acid, and vitamin D appears to have no significant effect on disease risk. Conversely, a high dietary inflammatory index has been linked to an increased risk of NMOSD, supporting the hypothesis that pro-inflammatory nutritional patterns may contribute to disease susceptibility [2]. Furthermore, patients with NMOSD have been shown to exhibit significantly lower nutritional status scores compared with healthy individuals, suggesting that overall nutritional inadequacy may play a role in the pathophysiology of the disorder [2].

These findings, while preliminary, fit into a plausible model linking oxidative stress, complement dysregulation, and astrocyte vulnerability—the mechanistic core of NMOSD. However, how these findings could be leveraged to develop interventions capable of producing long-term changes in disease progression remains unknown. To the best of our knowledge, no data are available on the possible role of diet as a modifier of NMOSD and MOGAD.

## 6. Physical Activity: Neuroprotection and Clinical Modulation

### 6.1. Exercise as a Protective Factor and Early Intervention

The World Health Organization defines physical PA as any bodily movement produced by skeletal muscles that requires energy expenditure, encompassing activities during work, transport, leisure, and exercise. In this manuscript, we distinguish between PA and structured exercise, as the two concepts are often used interchangeably despite their distinct meanings. PA refers to any bodily movement that results in energy expenditure. In contrast, structured exercise denotes a planned, repetitive, and purposeful subset of PA performed to improve or maintain physical fitness. This distinction is crucial in MS research and clinical practice, as structured exercise is the modality most consistently associated with neuroprotective and disease-modifying effects. Within the clinical and research context of MS, it is essential to distinguish general PA from structured exercise, the latter referring to planned, repetitive, and purposeful activity aimed at improving or maintaining physical fitness. In MS, structured exercise, whether aerobic, resistance, or combined training, has gained increasing attention not only as a therapeutic intervention but also as a potential modulator of disease trajectory in its early stages. Exercise should no longer be viewed solely as a rehabilitative measure; rather, it should be considered an integral part of early neuroprotective strategies, with the potential to delay disease progression and preserve CNS integrity from the earliest stages of MS pathology [154,155]. Beyond its physiological benefits, habitual PA is also being investigated as a functional biomarker. Objective monitoring of PA could help assess disability progression, suggesting that declining spontaneous activity levels may precede clinical worsening [156]. This positions PA not only as an intervention, but also as a surveillance tool in the early stages of MS. Supporting this view, higher levels of cardiorespiratory fitness were associated with greater preservation of thalamic functional connectivity in individuals with progressive MS [157]. This finding emphasizes the bidirectional relationship between brain health and physical capacity, reinforcing the utility of physical fitness as an indirect marker of neural reserve. Finally, the design and implementation of structured programs also play a key role in early intervention. For example, a lactate threshold-based multidisciplinary training protocol tailored for individuals with MS illustrates how exercise can be systematically integrated into clinical care, even for patients in the early or mild phases of the disease, laying the foundation for long-term neuroprotection and improved functional outcomes [158].

### 6.2. Mechanisms of Protection and Early Modulation

**Figure 2:**
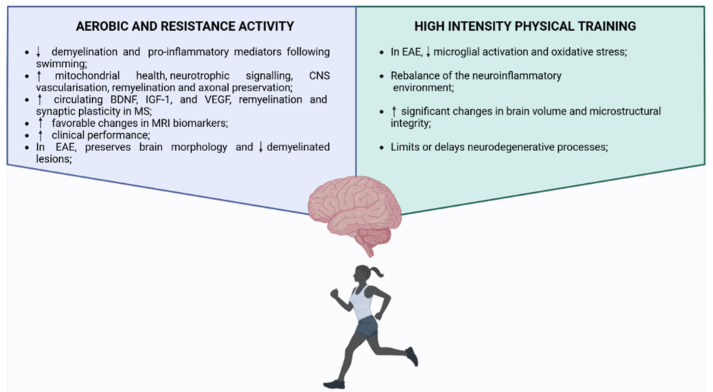
Effect on the CNS of aerobic and resistance activity and high intensity physical training in EAE and MS. ↑ indicates an increase, ↓ indicates a decrease.

In recent years, PA has gained recognition not only for its symptomatic benefits in MS, but also for its potential to modulate disease mechanisms at the neurobiological level (Figure 2).

Several studies [159,160] suggest that exercise may exert neuroprotective effects, promote remyelination, and enhance the structural and functional integrity of the CNS, especially when initiated early in the disease course. Mechanistically, exercise is thought to influence multiple pathways involved in neuroplasticity, mitochondrial function, angiogenesis, and inflammatory regulation. EAE studies have explored the neuroprotective effects of different forms of exercise, e.g., swimming. Animals undergoing regular swimming exhibited reduced demyelination and decreased levels of pro-inflammatory mediators compared with non-exercised controls. Physical training also led to improvements in body weight, motor performance, and muscle strength. Notably, rats were also injected with interferon-β and the beneficial effects of exercise were comparable to those achieved with interferon-β therapy, suggesting that swimming may serve as an adjunctive approach with significant immunomodulatory potential [161]. According to Lozinski and Yong [162], aerobic and resistance exercise can enhance mitochondrial health, upregulate neurotrophic signaling, and improve CNS vascularisation, creating an environment favorable to remyelination and axonal preservation. Their conceptual review highlights that these benefits may be significant when implemented before significant neurodegeneration occurs. Empirical evidence supports this theoretical framework. A systematic review and meta-analysis [160] confirmed that both aerobic and resistance exercise increase circulating levels of neurotrophic factors such as brain-derived neurotrophic factor (BDNF), insulin-like growth factor 1 (IGF-1), and vascular endothelial growth factor (VEGF), all of which are associated with remyelination and synaptic plasticity in MS. These molecular changes may underlie observed improvements in clinical and structural outcomes. High-intensity physical training also appears to modulate innate immune responses. In mouse model, Zaychik et al. [163] demonstrated that exercise reduced microglial activation and oxidative stress in the CNS, suggesting an exercise-induced rebalancing of the neuroinflammatory environment. These findings support the hypothesis that PA may limit or delay neurodegenerative processes. Human imaging studies reinforce these insights. In a randomized controlled trial (RCT), it was observed that high-intensity aerobic exercise led to significant changes in brain volume and microstructural integrity, particularly in regions vulnerable to MS-related damage [155]. Similarly, Savšek et al. [164] observed that aerobic training in individuals with MS resulted in favorable changes in MRI biomarkers, including white matter lesion volume and cortical thickness, alongside improvements in clinical performance. Preclinical models offer further insight into exercise-induced remyelination. In the cuprizone mouse model of EAE, aerobic training helped preserve brain morphology and reduce demyelinated lesions, suggesting that exercise may facilitate repair processes even in the absence of immunomodulatory drugs [159]. Finally, the concept of “MedXercise” was introduced, a framework in which exercise is systematically prescribed not only for symptomatic relief but also as a biological intervention to promote remyelination [165]. There is a need for further translating preclinical insights into clinical trials designed to assess structural repair and neurorestoration in MS [165]. Together, these findings will position PA as a multifaceted intervention capable of influencing both immune and neural components of MS pathophysiology. When introduced early, PA may not only support functional capacity but also contribute to long-term disease modification.

### 6.3. Aerobic vs. Resistance Training: Comparative Evidence

Among the various exercise modalities investigated in MS, both aerobic training (AT) and resistance training (RT) have demonstrated distinct and complementary benefits. Understanding the differential effects of these modalities is crucial for designing targeted interventions that optimize functional outcomes and disease modulation.

#### 6.3.1. Aerobic Training

AT, characterized by rhythmic, continuous movement that elevates heart rate and enhances cardiorespiratory endurance, has been widely studied in MS. One example is the lactate threshold training program developed by Amato et al. [158], which applied a multidisciplinary aerobic intervention tailored to individual physiological capacity. This program was shown to improve fatigue resistance, muscle function, and overall exercise tolerance in persons with MS, supporting the feasibility of structured AT even in clinical populations. Crucially, brain imaging studies have shown a link between AT and structural and functional brain changes. In a RCT, high-intensity AT led to improvements in gray matter volume and brain microstructure, including regions associated with motor and cognitive processing [155]. Similarly, AT resulted in reductions in lesion volume and improvements in cortical thickness, accompanied by gains in physical performance [164]. These findings underscore the neuroprotective potential of aerobic interventions. Support for AT as a remyelinating or anti-inflammatory stimulus also comes from preclinical models. Using the cuprizone mouse model of demyelination, AT helped preserve brain morphology and reduced demyelination, suggesting a direct impact on CNS repair mechanisms [159]. Plus, AT facilitates angiogenesis, mitochondrial function, neurotrophic factor release, and anti-inflammatory modulation, thereby creating an internal milieu conducive to remyelination and neuroprotection [162].

#### 6.3.2. Resistance Training

RT, which involves repeated contractions against external loads, targets muscular strength and functional independence, critical factors in MS-related disability. A recent meta-analysis confirmed that RT significantly enhances muscle strength and physical functionality in middle-aged individuals with MS, with modest effects on gait and fatigue [166]. These findings position RT as a key strategy in maintaining musculoskeletal integrity and functional reserve. Furthermore, a 12-week RT program in women with MS improved walking speed, grip strength, manual dexterity, and QoL, despite no measurable changes in oxidative stress biomarkers [167]. This could suggest that functional gains may occur independently of systemic biomarker shifts, possibly through neuromuscular adaptations. Additionally, a meta-analysis evaluating RT-based trials indicated that resistance exercise also increases the expression of BDNF and IGF-1 [160].

Beyond peripheral muscular benefits, RT induces several central adaptations relevant to MS pathophysiology. Evidence from neurophysiological studies shows that resistance training enhances corticospinal excitability, lowers motor activation thresholds, and increases neural drive [168], mechanisms that may counteract MS-related impairments in motor signal transmission and neuromuscular coordination. High-intensity RT has also been associated with reductions in serum neurofilament light chain, suggesting attenuation of axonal injury and a potential neuroprotective effect [169]. Moreover, RT facilitates type II muscle fiber hypertrophy, improves neuromuscular junction efficiency, and reduces central fatigue. These adaptations support improved mobility and functional capacity in individuals with MS. Ongoing clinical research further underscores this mechanistic relevance, with a dedicated trial investigating how progressive resistance training influences corticospinal excitability in MS [170].

Emerging evidence also suggests that RT may improve neuromuscular signaling and motor unit recruitment, which are often impaired in MS. It was also reported that high-intensity RT not only improved strength and functional capacity but also reduced serum levels of neurofilament light chain, suggesting a potential neuroprotective mechanism [171]. Similarly, it was demonstrated that task-oriented RT enhanced corticospinal excitability and walking performance, indicating improvements in motor signal transmission [172]. Recently it was emphasized that RT promotes type II muscle fiber recruitment, improves neuromuscular junction integrity, and reduces central fatigue, factors essential for preserving mobility in MS [173]. Moreover, RT can enhance maximal voluntary neural drive and reduce spasticity, indicating direct effects on motor control circuits [174]. These findings suggest that RT is not merely a peripheral intervention but may influence central neural processes, contributing to improved motor efficiency and neuroplasticity in MS.

Taken together, these findings highlight that resistance training is not solely a peripheral intervention aimed at increasing muscle strength. Rather, RT exerts multifaceted effects on both neuromuscular and CNS function, contributing to neuroplasticity, improved motor control, and potentially neuroprotective processes. Strengthening this section ensures a more balanced representation of RT relative to AE and underscores its relevance as a complementary modality in MS rehabilitation and disease modulation.

#### 6.3.3. Combined or Concurrent Training

Programs combining AT and RT, collectively referred to as concurrent training, have emerged as a promising strategy to address the multifactorial impairments associated with MS. This approach combines the cardiometabolic and anti-inflammatory benefits of AT with the neuromuscular, strength, and mobility benefits of RT. Rather than operating in parallel, these modalities may exert synergistic effects, creating a cumulative physiological impact greater than the sum of their effects alone. In a multidisciplinary protocol, [158] concurrent training was shown to enhance both muscle performance and aerobic capacity in individuals with MS, with significant reductions in fatigue perception and improvements in daily function. These findings align with another RCT [175] in women with MS which reported significant gains in strength, reductions in fatigue, and improvements in QoL following 12 weeks of combined endurance and resistance training. These benefits were partially maintained even after a 12-week detraining phase. Meta-analytic evidence further supports the use of concurrent training. A meta-analysis of 12 RTC, authors concluded that combined exercise training exerted the most potent effect on health-related QoL, outperforming both aerobic- and resistance-only interventions [176]. Concurrent programs offer additive or synergistic value, particularly in addressing the broad spectrum of symptoms associated with MS. Importantly, the Early Multiple Sclerosis Exercise Study brought attention to the potential disease-modifying role of concurrent training when initiated in the early stages of MS [177]. The structured intervention combined progressive aerobic and resistance elements over 24 weeks, targeting individuals recently diagnosed with RRMS. Improvements in cardiorespiratory fitness, walking endurance, muscle strength, and neurocognitive measures, as well as preservation of brain volume and microstructural integrity on MRI were observed [177]. These findings suggest a potential neuroprotective effect, particularly when the intervention is initiated before irreversible neuroaxonal loss occurs. Physiologically, concurrent training engages multiple adaptive pathways simultaneously. Aerobic exercise stimulates mitochondrial biogenesis, angiogenesis, and anti-inflammatory cytokine profiles, while resistance training increases motor unit recruitment, muscle fiber hypertrophy (mainly type II fibers), and neuromuscular efficiency. Studies suggest that when appropriately combined, with adequate recovery and intensity modulation, these adaptations may complement rather than interfere with one another. Moreover, concurrent training may be particularly advantageous for addressing the heterogeneity of symptoms seen in MS. For instance, patients with predominant fatigue may benefit from aerobic components that modulate central energy metabolism. In contrast, those with early mobility loss or muscular atrophy may see faster benefits from resistance-based components [177]. This flexibility enhances patient adherence and allows for personalized rehabilitation planning based on phenotype, disease stage, and comorbid conditions. Despite these promising outcomes, concurrent training in MS remains understudied relative to single-modality interventions. Key questions remain regarding the optimal frequency, sequence (e.g., resistance before or after aerobic work), dosage, and progression strategies. Furthermore, while EMSES has paved the way, there is a need for larger, multicenter, and longer-term trials with neuroimaging, immunological, and functional endpoints to fully confirm its disease-modifying potential.

### 6.4. Timing Matters: Why Early Is Better

The timing of exercise initiation in MS is increasingly recognized as a crucial factor in maximizing its neuroprotective potential. Growing evidence indicates that engaging in structured physical training early in the disease course, preferably at the clinically isolated syndrome (CIS) or early RRMS stage, may provide benefits beyond symptom management, potentially influencing disease progression and neural integrity. Evidence suggests that exercise should be integrated into the care pathway from the earliest stages, not only as a rehabilitative tool but also as a proactive intervention aimed at preserving brain reserve [154]. Early MS is characterized by a period of increased neuroplasticity, during which the capacity for remyelination and adaptive immune regulation remains relatively preserved. Exercise introduced during this phase may boost these innate repair mechanisms. This rationale is supported by findings from the EMSES study that specifically targeted individuals in the early stages of MS and involved a 24-week structured concurrent exercise program [177]. Results showed improvements in physical function and cardiorespiratory fitness, along with MRI-based evidence of preserved brain volume and microstructural integrity. These findings suggest that early exercise may reduce disease-related neurodegeneration and enhance CNS resilience [177].

Additionally, Stuart et al. [156] proposed that PA monitoring could serve as a functional indicator of disease status. In their study, declining PA levels were associated with increased disability progression, reinforcing the idea that reduced activity may be both a consequence and predictor of early neurofunctional decline. This highlights the value of using PA as both an intervention and a marker to guide early therapeutic strategies. Conceptually, the “use it or lose it” principle applies strongly to MS. As Lozinski and Yong [162] note, delaying PA interventions may result in missed opportunities to stimulate angiogenesis, neurotrophic signaling, and mitochondrial health, all of which are critical to maintaining CNS integrity. Once irreversible neuroaxonal loss occurs, these pathways become less responsive, and the effects of exercise are likely to diminish.

### 6.5. Physical Activity in NMOSD and MOGAD

Reduced PA during adolescence could also be involved in NMOSD risk [2]. Moreover, several studies on rehabilitation exercise in acute spinal cord injury have shown that early rehabilitation can facilitate recovery of motor control and activate residual neural networks, thereby improving movement and overall function. Spinal cord injury may also be considered comparable, in some aspects, to the inflammatory myelitic damage observed in the acute phases of an NMOSD attack [178].

However, few data on the impact of PA on NMOSD and MOGAD are available in the literature, as the diseases are rare and emerging. It should be noted that most evidence supporting the benefits of PA derives from studies conducted in MS, and its extension to NMOSD and MOGAD remains largely speculative. Differences in underlying immunopathology, disease course, and disability patterns limit direct extrapolation, highlighting the need for disease-specific studies to determine whether similar exercise-mediated effects are applicable to antibody-mediated demyelinating disorders. Patients undergoing PA after a relapse usually experience a favorable outcome, particularly with a multidisciplinary and tailored intervention [179]. However, no specific data on different protocols, long-term benefits, and the influence of PA are available, even though we can speculate that the benefits highlighted for MS patients may also apply to NMOSD and MOGAD patients. PA could be beneficial for pain, bladder dysfunction, cognitive impairment and fatigue in NMOSD [180]. However, from a biological point of view, animal studies showed that PA promotes astrocyte proliferation and morphological changes, enhances their neuroprotective role via increased glutamate uptake and upregulation of neurotrophic factors [181,182]. PA can enhance metabolic activity, thereby supporting mitochondrial function in neurons and reducing damage caused by oxidative stress. Aerobic training was shown to improve mitochondrial quality in astrocytes in an animal model of Alzheimer’s disease, and regular moderate aerobic exercise has been found to reduce pain-related signs in animal models of NMOSD by increasing endogenous opioid content in the brainstem [180]. Moreover, regular PA was found to reduce neuronal apoptosis and attenuate cognitive decline in mouse model of Alzheimer’s disease, by upregulating the astrocytic transporter Slc2a1, which mediates glucose transport across the BBB and between glial cells (a process essential for maintaining proper brain energy metabolism) [183]. Finally, studies in MS have shown that regular aerobic and moderate-intensity exercise can modulate the systemic inflammatory response, including a reduction in interleukin-6 (IL-6) levels at rest and after exercise, promoting an anti-inflammatory environment [184]. IL6 is now known to be a pivotal cytokine in the pathogenesis of NMOSD and MOGAD as well [185,186]. Although no specific data are currently available for NMOSD and MOGAD, we could speculate that this effect observed in MS may also apply to these conditions, given the central role of IL-6 in their pathogenesis.

## 7. Discussion

The comprehensive analysis presented in this narrative review supports the paradigm shift toward integrating lifestyle interventions, namely diet and physical exercise, as adjunctive disease modifiers from early stages of CNS inflammatory demyelinating diseases, particularly MS. Diet and PA converge on shared mechanisms, including modulation of Th17/Treg balance, oxidative stress, neurotrophic signaling, and BBB integrity, supporting a unified model in which lifestyle interventions jointly influence immune system and CNS rather than acting as isolated risk factors [3,4,187,188]. Starting with diet, differences in micro- and macronutrients (glucose, salt, vitamin A and D, fat, and caloric intake) observed in EAE, impact T cells and B cells and microglia functions linked to pro-inflammatory activity in MS [81]. Data on neurodegenerative processes remain scarce, though intriguing [3,81,99,134].

When considering specific diets, the majority of studies observed improvements in patients’ reported outcomes, such as fatigue; however, iCR and KD also reduced pro-inflammatory and neurodegenerative biological markers in patients [123,126], and MD and MIND improved disability outcomes [4,129,138]. MD, MIND, KD, and iCR were associated with favorable outcomes, including reduced disability progression, relapse rates, cognitive impairment and brain atrophy, although observational and cross-sectional designs limit causal inference [4,117,129,131,132,135,145]. Comparisons between regimens are often lacking, and the long-term feasibility of regimens remains unclear, as RCTs are typically 6 to 12 weeks long [126]. Adherence to MD has been evaluated longitudinally, but in small samples and without a control group [4]. These aspects can serve as a starting point for future research. Neurologists should also become accustomed to investigating patients’ dietary habits from early disease stages, and incorporate structured lifestyle counseling into routine neurological care, encouraging closer collaboration with rehabilitation specialists, dietitians, and exercise professionals.

Several limitations occurred in our results. A major methodological constraint is the high heterogeneity of interventions and outcome measures across studies, which precludes definitive conclusions regarding optimal dosage or modality for long-term disease modification. For diet, most positive results rely on observational data or clinical trials primarily reporting subjective endpoints such as fatigue and QoL, with insufficient evidence from RCTs to conclusively modify disability as measured by the EDSS. Furthermore, relapse rate data often lack adjustment for concurrent DMTs adherence, potentially confounding the true impact of nutritional habits. Plus, studies are often cross-sectional, use small sample sizes, or have short follow-up periods, limiting the generalizability of the results. Finally, many data rely on animal models, which may not necessarily translate to human patients. Moreover, when considering studies on singular micro- or macronutrients, such as vitamin D, ethnicity, geographic, and genetic background also need to be taken into consideration. As an example, while strong associations between low vitamin D levels and disease risk or activity have been consistently reported in predominantly Caucasian MS cohorts, findings in NMOSD and in non-European populations are more heterogeneous. Differences in genetic background, skin pigmentation, sunlight exposure, dietary habits, and vitamin D metabolism may contribute to these discrepancies and should be carefully considered when interpreting existing data and extrapolating potential clinical implications. Therefore, we could speculate that different practical lifestyle interventions should be applied based on our patients’ ethnicity and geographic location.

Physical exercise also modulates core pathological pathways, including reducing oxidative stress, promoting anti-inflammatory cytokine profiles, and inducing beneficial shifts in effector T cell subsets [125,189,190,191]. Moreover, we highlighted the converging evidence demonstrating the neuroprotective potential of structured exercise in MS. The accumulating evidence supporting the role of exercise in MS points to a paradigm shift: from viewing PA solely as a rehabilitation strategy to recognizing it as a potential disease-modifying and neuroprotective intervention. Our narrative review highlights that both aerobic and resistance training, can improve neurological function, cognitive performance, and CNS integrity, especially when introduced early in the disease course [155,166,192]. From a clinical perspective, this necessitates a more proactive approach to exercise prescription in MS from neurologists. Tailored programs that combine aerobic endurance, muscular strength, and neuromuscular coordination should be offered alongside pharmacological therapy—not as an adjunct for symptomatic relief, but as an integrated component of early intervention strategies [154,177]. These programs should ideally be individualized, supervised, and adjusted to disease stage, fatigue level, and comorbidities. Despite promising results, however, several important research gaps remain. First, while multiple studies have demonstrated functional and structural improvements following exercise, few have directly assessed long-term effects on relapse rate, disability progression, or lesion burden. Most trials focus on short-term outcomes and are underpowered to detect disease-modifying effects comparable to pharmacologic agents. Second, the biological mechanisms by which exercise may exert neuroprotective or remyelinating effects are still incompletely understood. While increases in BDNF and IGF-1 and reductions in neurofilament light chain have been reported [160,188], results across studies remain inconsistent, and no routinely used biomarkers are available [193]. More standardized protocols and biomarker panels are needed to validate these outcomes. Third, evidence on RT is still less robust than that on aerobic training, particularly regarding its effects on corticospinal excitability, neuromuscular signaling, and motor unit recruitment [166]. This represents a crucial area for further investigation, especially given RT’s potential to preserve independence and prevent deconditioning in progressive MS. Moreover, there is a lack of longitudinal and multicenter studies assessing the durability of training-induced adaptations. Few trials extend beyond 6–12 months, limiting our understanding of whether continued training translates into clinically meaningful protection over time. Lastly, individuals in the prodromal or very early stages of MS, such as those with CIS or RIS, remain underrepresented in exercise trials. These populations may benefit the most from early intervention, yet are often excluded.

To note, combined RCTs comparing different types of exercises and diets together are still missing, possibly representing an interesting new field to be explored. Plus, the magnitude and direction of lifestyle-related effects are likely influenced by interindividual factors such as genetic background, microbiome composition, metabolic status, and heterogeneity in disease course, reinforcing the need to interpret lifestyle interventions within a personalized medicine framework and to implement future research. Future trials should move beyond descriptive associations and adopt standardized intervention protocols, stratification by disease stage and treatment status, and longitudinal designs capable of assessing true disease-modifying effects.

Finally, the large knowledge gap relies on results for NMOSD and MOGAD. These asymmetries, compared with MS, delineate priority areas for future research. Specific long-term data on lifestyle modification as a disease modifier are virtually absent for NMOSD and MOGAD. While plausible mechanistic links exist, such as the association between diet, oxidative stress, and AQP4 vulnerability in NMOSD, or the potential role of IL-6 modulation through exercise, these remain extrapolations from MS or preclinical models. Future research must address these gaps through larger, longitudinal, multicenter trials that utilize standardized protocols, comprehensive biomarker panels, and rigorous assessment of functional and structural outcomes across the entire spectrum of CNS inflammatory demyelinating diseases. Next steps involve translating these promising mechanistic insights into personalized, evidence-based clinical recommendations.

## 8. Conclusions

In conclusion, diet and exercise are not only involved in the development of CNS inflammatory disorders but also have potential as additional disease modifiers to be targeted alongside DMTs. Lifestyle interventions should be considered complementary to disease-modifying therapies and tailored according to disease phenotype, comorbidities, metabolic profile, and ongoing pharmacological treatment. More targeted research is needed to address current limitations. Addressing these gaps will be essential to integrating lifestyle interventions into standard care pathways, thereby improving both short- and long-term neurological outcomes.

## Figures and Tables

**Table 1:** Summary table of the biological and clinical effect of different diets in MS.

Ref	Diet Types	Clinical Study Design	Biological Effects	Clinical Effects
[127]	MD	Meta-analysis of 12 RCTs (608 patients)		Improvements in physical and mental QoL ↓ Fatigue scores High/moderate risk of bias, small sample sizes
[128]	MD	Meta-analysis of 5 RCTs (540 patients)		↓ Body mass index Non-significant marginal ↓ in fatigue
[4,129,130,131,132]	MD	5 Observational studies (cross-sectional or retrospective studies + one prospective longitudinal study of 1 year)		Adherence to MD is inversely associated with EDSS, MSSS, and Multiple Sclerosis Walking Scale Higher MD adherence scores mitigate the negative impact of disease duration
[133]	MD	Observational study (478 Iranian patients)		No association between self-reported adherence to MD score and disability.
[137]	MIND	Case–control longitudinal study (12-week dietary intervention)	↓ BDNF ↓ Biomarkers of oxidative stress	↓ Fatigue scores ↑ Physical QoL
[138]	MIND	Interventional study (8-week dietary intervention)	↑ Serum level of total antioxidant compared to baseline	
[123,124]	KD	Prospective study (6-month dietary intervention)	↓ Leptin ↑ Adiponectin ↓ NfL	↓ Self-reported fatigue and depression scores ↓ EDSS ↓ 6-Minute Walking Test ↓ Nine-Hole Peg Test
[125]	iCR	RCT (8-week 5:2 iCR compared to standard diet)	↓ Memory T cell subsets and Th1 ≠ in glycerophospholipid metabolism products	
[126]	iCR	RCT (12-week 5:2 iCR compared to standard diet)	↓ Naive CD4+ T cells ↓ Th1 cells Treg were more active ↓ Leptin	Significant improvement in the Symbol Digit Modality Test Score
[127]	Paleolithic diets	Meta-analysis of 12 Randomized Trials (608 patients)		Improvements in physical and mental QoL ↓ Fatigue

↑ indicates increase; ↓ indicate decrease.

## Data Availability

No new data were created or analyzed in this study. Data sharing is not applicable to this article.

## Abbreviations

The following abbreviations are used in this manuscript:

APC	antigen-presenting cells
BDNF	Brain-Derived Neurotrophic Factor
BBB	Brain blood barrier
CNS	Central Nervous System
CIS	Clinically Isolated Syndrome
DASH	Dietary Approaches to Stop Hypertension
DMTs	disease-modifying treatments
EBV	Epstein–Barr Virus
EDSS	Expanded disability Status Score
EAE	Experimental Autoimmune Encephalomyelitis
HLA	Human Leukocyte Antigen
IFG-1	Insulin-like Growth Factor 1
iCR	Intermittent caloric restriction
KD	Ketogenic Diet
MD	Mediterranean Diet
MIND	Mediterranean–DASH Intervention for Neurodegenerative Delay
MOGAD	Mog Associated disorder
MS	Multiple Sclerosis
NMOSD	Neuromyelitis optica spectrum disorder
PLP	proteolipid protein
PA	Physical Activity
QoL	Quality of Life
RR	Relapsing Remitting
RT	Resistance training
RCT	Randomized controlled trial
TAC	total antioxidant capacity
